# O7.4. SYSTEMATIC REVIEW, META-ANALYSIS AND META-REGRESSION OF PREDICTORS OF PLACEBO RESPONSE IN ACUTE SCHIZOPHRENIA

**DOI:** 10.1093/schbul/sby015.233

**Published:** 2018-04-01

**Authors:** Claudia Leucht, Anna Chaimani, Stefan Leucht

**Affiliations:** 1 Technical University of Munich; 2 Paris Descartes University

## Abstract

**Background:**

The drug-placebo differences (“effect sizes”) in acute phase, randomised, double-blind trials have become smaller and smaller over the decades. In a recent meta-regression analysis, it had been shown that the degree of placebo response is the strongest predictor of drug-placebo differences. Thus, the open question now was what the predictors of placebo response are.

**Methods:**

Placebo-controlled, randomised, double-blind trials that compared any licensed antipsychotic drug with placebo were searched in multiple electronic databases, the website of the Food and Drug Administration and the clinical trial database ClinicalTrials.gov. The mean change from baseline of the PANSS or the BPRS total score from baseline to endpoint in the placebo-groups was extracted from each identified trial. The outcome was the degree of placebo response measured by the BPRS or PANSS change from baseline to endpoint. 24 patient-, and study design related parameters were analysed as potential predictors of placebo response in univariate and multivariate meta-regression analyses.

**Results:**

Of 167 included RCTs 99 provided the necessary data. In univariate analyses more recent publication year, larger sample size (total number of participants and sites), use of PANSS rather than the BPRS, studies conducted outside the US or mixed, shorter wash-out phases and shorter study duration, lower participant mean age and lower mean duration of illness were associated with higher placebo-response.

**Discussion:**

This meta-regression included approximately two times more studies than previous attempts to resolve this issue and it is therefore the to date by far largest analysis of this kind. Multiple potential moderators of placebo response were identified. Importantly, these moderators of placebo response were not identical with those identified in a previous analysis1 as significant moderators of drug-placebo differences in the same dataset. Thus, different factors appear to play a role in this complex area.

**References:**

1. Leucht S, Leucht C, Huhn M, Chaimani A, Mavridis D, Helfer B, Samara M,Rabaioli M, Bächer S, Cipriani A, Geddes JR, Salanti G, Davis JM. Sixty Years of Placebo-Controlled Antipsychotic Drug Trials in Acute Schizophrenia: SystematicReview, Bayesian Meta-Analysis, and Meta-Regression of Efficacy Predictors. Am J Psychiatry. 2017;174(10):927–942.

